# Enabling Survival of Transplanted Neural Precursor Cells in the Ischemic Brain

**DOI:** 10.1002/advs.202302527

**Published:** 2023-10-22

**Authors:** Zhifu Wang, Danyi Zheng, Ye Sing Tan, Qiang Yuan, Fang Yuan, Su‐Chun Zhang

**Affiliations:** ^1^ Program in Neuroscience & Behavioral Disorders, GK Goh Centre for Neuroscience Duke‐NUS Medical School Singapore 169857 Singapore; ^2^ Department of Neuroscience Department of Neurology Waisman Center University of Wisconsin‐Madison Madison WI 53705 USA

**Keywords:** CCR5 antagonists, fibrinogen, ischemic strokes, neural regenerations, transplantations

## Abstract

There is no effective therapy for ischemic stroke following the acute stage. Neural transplantation offers a potential option for repairing the ischemic lesion. However, this strategy is hindered by the poor survival of the neural precursor cells (NPCs) that are transplanted into the inflammatory ischemic core. Here, a chemical cocktail consisting of fibrinogen and maraviroc is developed to promote the survival of the transplanted NPCs in the ischemic core of the mouse cerebral cortex. The grafted NPCs survive in the presence of the cocktail but not fibrinogen or maraviroc alone at day 7. The surviving NPCs divide and differentiate to mature neurons by day 30, reconstituting the infarct cortex with vascularization. Molecular analysis in vivo and in vitro shows that blocking the activation of CCR5 on the NPCs protects the NPCs from apoptosis induced by pro‐inflammatory factors, revealing the underlying protective effect of the cocktail for NPCs. The findings open an avenue to enable survival of the transplanted NPCs under the inflammatory neurological conditions like stroke.

## Graphical abstract

A chemical cocktail consisting of fibrinogen and CCR5 antagonist maraviroc supports the survival and maturation of neural precursors that are transplanted into the ischemic stroke cavity by blocking the inflammatory cascade, enabling the reconstitution of the damaged central nervous system by grafted neural cells.

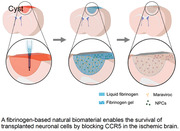

## 1 Introduction

Ischemic stroke results from a sudden reduction in cerebral blood flow. It has afflicted ∼25% people over their lifetime, accounting for almost 5% of all disability‐adjusted life‐years and 10% of all deaths worldwide.^[^ ^1^ ^]^ Current treatment is reperfusion during the acute stage of stroke. There is no effective treatment beyond the acute stage. There is, therefore, an unmet need to develop a treatment for ischemic stroke.

Stem cell‐based approaches hold promise as cell therapies may protect the injured neurons from further damage and/or replace the lost neurons. Transplantation of non‐neural cells, such as mesenchymal stem cells (MSCs), has been initiated in clinical trials. These studies have shown the protection of neurons in the penumbral area, an area bordering the ischemic site and the healthy brain region, by modulation of inflammation or angiogenesis but not neuronal replacement.^[^ ^2^, ^3^ ^]^ Since the human brain has a very limited capacity to regenerate and MSCs usually do not produce neurons, it is desirable to have neural cells as a source for cell therapy. Indeed, transplantation with neural precursor cells (NPCs) has demonstrated that they can mature to become functional neurons and potentially integrate into host brain circuitry in animal models of neurological conditions, e.g., stroke, spinal cord injury (SCI), and Parkinson's disease (PD).^[^ ^4^, ^5^, ^6^, ^7^, ^8^ ^]^ In animals with ischemic stroke, the ischemic/infarct site forms a cavity that is walled off by glial scars and filled with inflammatory cells and secretions. Such an environment is hostile to transplanted NPCs. Hence, most of the studies to date involve transplantation of cells into the penumbra.^[^ ^8^, ^9^, ^10^, ^11^ ^]^ The advantage of transplanting into the penumbra avoids the hostile environment in the cavity. Nevertheless, it creates an additional injury to the healthy brain region. It also leaves the cavity unfilled, making it difficult to reconstruct the damaged circuit.

NPC transplantation into the cavity would fill the gap, replace the lost neural cells, and potentially reconnect the disrupted circuitry. The first and key step is to enable the survival of the transplanted NPCs. Efforts have been made to improve the survival of NPCs that are transplanted into the ischemic core, including overexpressing Small Ubiquitin‐like Modifier (SUMO), hypoxic treatment, co‐transplantation with non‐neuronal cells,^[^ ^12^, ^13^, ^14^, ^15^ ^]^ hydrogels crosslinked with growth factors like bone morphogenetic proteins (BMP4), brain‐derived neurotrophic factor (BDNF), and laminin derived motif (IKVAV) based biomaterials.^[^ ^16^ ^]^ In particular, hydrogels possess anti‐inflammatory action and can be resorbed by the tissue. Several studies demonstrated that hyaluronan/methylcellulose exhibits anti‐inflammatory properties by reducing IL‐1α levels in the central nervous system after stroke and spinal cord injury.^[^ ^16^, ^17^, ^18^, ^19^ ^]^ The hydrogels may also be modified to modulate immune response and promote angiogenesis,^[^ ^20^ ^]^ potentially promoting the survival and differentiation of transplanted NPCs. When encapsulated in hydrogels, NPCs transplanted into the stroke cavity can survive with limited proliferation for 2 weeks.^[^ ^21^ ^]^ An anti‐inflammatory polarising effect of hydrogel on infiltrating microglia could indicate the potential for inflammatory reprogramming of the stroke lesion,^[^ ^17^, ^21^, ^22^, ^23^ ^]^ which could contribute to neural regeneration after NPC transplantation. These methods help the survival but fail to fill and reconstitute the damaged brain due to limited number of surviving cells. The poor cell survival even in the presence of neurotrophic support relative to that in other neurological models such as the 6‐OHDA induced Parkinson's disease models,^[^ ^5^, ^24^, ^25^ ^]^ led us to hypothesize that modification of the hostile ischemic environment, especially the inflammatory milieu, is critical for promoting the NPC survival.

Whether and how the chronic neuroinflammation triggers death of the grafted NPCs remains unclear. Recent studies report that upregulation of chemokine receptor (CCR)5 reduces the survival of dendritic spines and triggers pyroptosis of mature neurons through the PKA/CREB pathway in neurological diseases.^[^ ^26^, ^27^, ^28^ ^]^ In the adult central nervous system (CNS), CCR5 is highly expressed on microglia compared to neurons, astrocytes, and oligodendrocytes but its expression on mature neurons is significantly upregulated under neurological conditions, including stroke, traumatic brain injury (TBI), and Alzheimer's disease (AD).^[^ ^27^, ^28^, ^29^, ^30^, ^31^ ^]^ In the present study, we found that CCR5 was highly expressed on NPCs and its expression was dramatically upregulated by its ligands, killing the NPCs even in vitro. We hence developed a cocktail consisting of an FDA‐approved CCR5 antagonist maraviroc,^[^ ^32^, ^33^ ^]^ and fibrinogen that forms a gel to slow the dilution of maraviroc to enable the survival of NPCs. In the presence of the cocktail, the NPCs grafted into the ischemic core, survived and subsequently differentiated to neurons, which reconstitutes the collapsed cortex.

## 2 Results

### 2.1 Combination of Fibrinogen and CCR5 Antagonist Protects Grafted NPCs from Apoptosis in the Ischemic Core

**Figure 1:**
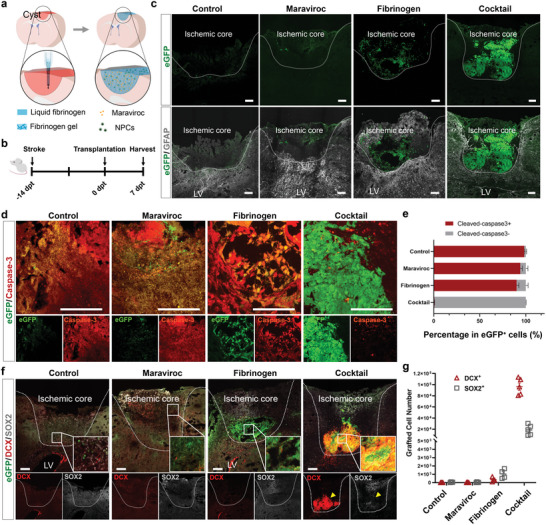
Survival of grafted NPCs in the ischemic core. a) Schematic showing the process of NPC transplantation with fibrinogen and maraviroc. b) Experimental procedures for the induction of ischemic stroke, cell transplantation, and tissue harvest for analysis. c) Immunostaining for GFAP in stroke mice transplanted with NPCs in the present of a‐CSF, maraviroc, fibrinogen or the cocktail at day 7 post transplantation (7 dpt), showing the GFP+ grafted cells (green) in the cavity (marked by dash outline) and GFAP+ glial scar surrounding the cavity. Scale bar, 200 µm. d) Cortical slices showing the immunoreactivity of cleaved‐caspase3 (red) in grafted cells at 7 days after transplantation. Separate fluorescent channels shown below. Scale bar, 100 µm. e) Quantification of the proportion of cleaved‐caspase3+ cells in GFP+ cells. *n* = 4 mice per group. Data are mean ± SEM. f) Immunostaining for DCX and SOX2 in stroke mice transplanted with NPCs at 7 days after transplantation. Yellow arrowhead indicates surviving grafts. Scale bar, 200 µm. g) Number of DCX+ or SOX2+ cells among GFP+ cells in different groups. Dotted lines outline the ischemic core in (c) and (f). *n* = 5 mice for the cocktail group, *n* = 4 mice for all other three groups. Data are mean ± SEM.

We are targeting cell therapy for chronic stroke, as spontaneous recovery is rare at this stage. During the chronic phase of stroke, the infarct area forms a cyst or cavity that is walled off by glial scar tissues and filled with inflammatory cells and their secretions.^[^ ^34^, ^35^, ^36^, ^37^ ^]^ Such an environment not only lacks physical and nutritional supports for but also exerts inflammatory effects on grafted NPCs. To modify the hostile environment in the infarct core, we developed a cocktail comprising Maraviroc (5 mg mL^−1^) and fibrinogen (9 mg mL^−1^). Maraviroc is an FDA‐approved CCR5 inhibitor that specifically blocks the binding between CCR5 and its ligands. To slow the release of Maraviroc, we mixed it with a hydrogel. We chose fibrinogen (9 mg mL^−1^) as it retains a soluble state, making it easy for transplantation. Upon injection, the fibrinogen mixed with the endogenous thrombin released during surgery and became a gel. Such an injectable gel not only stabilized the grafted cells but also slowed the dilution of maraviroc, as indicated by the release profile of Maraviroc (**Figure** **1a**; Figure S1a, Supporting Information). We assessed the effect by transplanting eGFP‐labeled cortical NPCs (differentiated from human embryonic stem cells [hESCs], the eGFP‐H9 hESC line, for 50 days, Figure S1c–f, Supporting Information) directly into the ischemic core at two weeks after stroke in the absence or presence of maraviroc, fibrinogen, or the cocktail and then measured the viability of the grafted cells one week later (Figure 1b,c). The ischemic stroke was induced by photothrombosis in the cerebral cortex of SCID mice in which the ischemic cavity was surrounded by GFAP+ and S100β+ glial scar at day 14 (Figure 1c; Figure S1b–h, Supporting Information) and the cortex collapsed at day 30 without treatment (Figure S1i, Supporting Information). The ischemic injury induced by photothrombosis presents a relatively uniform size at a similar location, offering a consistent model for assessing the efficacy of cell replacement therapy.

Grafted cells, identified by GFP, were observed in the stroke cavity in the presence of fibrinogen or the cocktail but few or no GFP+ cells in the presence of maraviroc or the cell‐only group (Figure 1c; Figure S2a, Supporting Information). Immunostaining for cleaved‐caspase3 revealed strong fluorescence in the control, maraviroc, and fibrinogen groups with diffuse staining in the control and maraviroc groups and discrete staining in individual GFP+ cells in the fibrinogen group (Figure 1d,e), suggesting that the grafted (GFP+) cells in the control and maraviroc groups are dead (fragmented) and the individual GFP+ cells in the fibrinogen group are dying. In contrast, few GFP+ cells were positive for caspase in the cocktail group (Figure 1d,e). Thus, the transplanted NPCs survive in the presence of the cocktail.

Stereological quantification of the GFP (overlay on DAPI‐labelled nuclei) showed the presence of 1.2 × 10^5^ cells in the cocktail group (Figure 1f,g). Over 26% and 81% of grafted cells were SOX2+ and DCX+, respectively, indicating that most of NPCs are at the immature stage and begin to differentiate to neurons (Figure 1f,g). Immunostaining for Ki67, a marker for cell proliferation, revealed that ≈11% of the GFP+ cells were Ki67+ in the cocktail group, but few in the other three groups (Figure S2b,c, Supporting Information). Together, the results show that fibrinogen or maraviroc alone is insufficient to improve the survival of grafted NPCs; combination of them supports the survival of the NPCs that are transplanted into the ischemic core.

### 2.2 Surviving NPCs Become Mature Neurons

**Figure 2:**
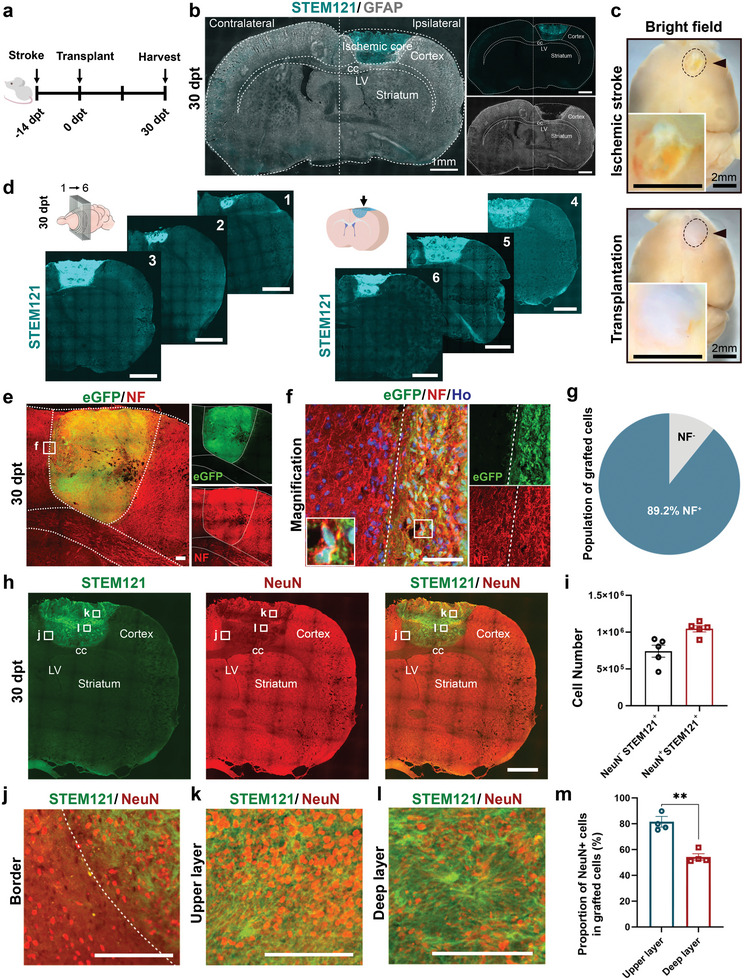
Maturation of the grafted neurons at 30‐day after transplantation. a) Schematic showing the experimental strategy. b) Overview of transplanted human cells (labelled by STEM121) in the ischemic core (surrounded by GFAP+ glial scar) of the cortex in stroke mice transplanted with the cocktail at 30 days post transplantation (30‐dpt). Separate channels on the right. Scale bar, 1 mm. c) Whole‐mount view of brains from mice with (bottom panel) or without (top panel) transplantation. Dotted lines and black arrowheads indicate the injured or transplant site. Scale bar, 2 mm. d) Serial coronal slices show STEM121+ cells fill up the stroke cavity at 30‐dpt. Scale bar, 1 mm. e,f) Immunostaining for neurofilament (NF) showing expression of NF in grafted cells (GFP+ cells) at 30‐dpt. Magnified images (f) show GFP+ cells are NF positive. Scale bar, 200 µm in (e), 100 µm in (f). g) Quantification of the percentage of NF+ cells in GFP+ cells. *n* = 4 mice. Data are mean ± SEM. h) Immunostaining for STEM121 and NeuN showing grafted cells differentiate to mature neurons at 30‐dpt. Scale bar, 1 mm. i) Number of GFP+ NeuN+ or GFP+ NeuN‐ cells. *n* = 5 mice. Data are mean ± SEM. j–l) Images of areas indicated in (h) showing grafted cells at the border (j), upper layer (k), and deep layer (l). Scale bar, 100 µm. m) Quantification of the percentage of NeuN+ cells in grafted cells in the upper layer and deep layer. *n* = 4 mice. Data are mean ± SEM. *p* = 0.0013. ^**^ *p* < 0.01. LV, lateral ventricle. cc, corpus callosum.

The milieu in the ischemic core is generally inhibitory to the differentiation of grafted NPCs.^[^ ^38^ ^]^ To determine if the transplanted NPCs survive for a longer term and what they become, we assessed the transplanted brains and the number and fate of the transplanted NPCs at 30 days post transplantation (**Figure** **2a**). Grossly, the brains from the sham (stroke without transplantation), control, maraviroc and fibrinogen groups displayed the collapsed cortex with no or few GFP+ cells in the ischemic area (Figure S3a, Supporting Information), whereas those from the cocktail group showed a smooth surface that is similar to the contralateral side (Figure 2b,c). The stroke cavity is walled off by glial scar tissues; hence it can be traced by using GFAP and/or S100β staining. We observed that GFP labelled, transplanted cells were confined to the ischemic core, surrounded by GFAP+ and S100β+ glial scar (Figure 2b), suggesting that the grafted neurons did not migrate to the peri‐infarct area. Strikingly, the transplanted GFP+ cells filled the entire stroke cavity at one‐month post transplantation (Figure 2b,c). Serial coronal brain sections further revealed that the grafted cells, confirmed by positive staining for the human specific marker STEM121, filled up the infarct area (Figure 2d), and <0.4% of GFP+ cells express Ki67 (Figure S3b,c, Supporting Information), indicating that transplanted NPCs largely ceased proliferation by one month without overgrowth.

Immunostaining for mature neuronal markers indicates that over 89% of the transplanted cells express neurofilament (NF) (Figure 2e–g). Similarly, 56% of the grafted human (STEM121+) cells were positive for NeuN, another mature neuronal marker (Figure 2h,i). The proportion of NeuN+/ STEM121+ cells appeared gradient, with more NeuN+ cells in upper layers and the edge of the stroke cavity than the center in grafts (Figure 2j–m). About 9% of GFP+ cells expressed SOX9, a marker for astroglia or their progenitors (Figure S3d,e, Supporting Information). Although the neurons were localized to the ischemic cavity, their neurites, indicated by positive staining for the human marker STEM121 and glutamatergic neuron marker vGluT1, grew into the undamaged region adjacent to the site of injury (Figure S4a,b, Supporting Information). Furthermore, the STEM121+ neurites co‐expressed a presynaptic marker synapsin and a post‐synaptic marker PSD95 (Figure S4c, Supporting Information), demonstrating that the grafted cells develop to mature neurons and form synapses with host neurons. Together, these results indicate that the human NPCs developed to mature neurons in the presence of the cocktail within 30 days.

To assess if the cell transplantation contributes to functional improvement, we conducted behavioral tests, including the rotarod test and grid‐walking test. While the latency to fall in the rotarod test showed improvement in the cocktail group (Figure S5a, Supporting Information), the grid‐walking test did not exhibit significant difference between the cocktail groups and the control groups (Figure S5b, Supporting Information). Hence, a partial functional recovery is achieved at 1 month after NPC transplantation with the cocktail.

### 2.3 Successful Transplantation is Associated with Mitigated Glial Reaction and Restored Vascularization

**Figure 3:**
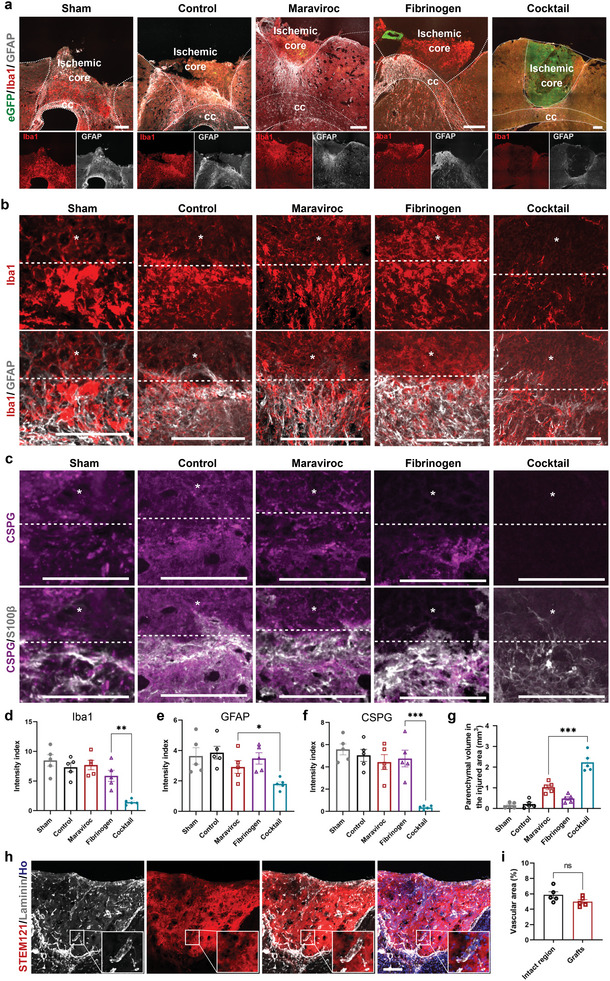
Successful transplantation is associated with mitigated glial reaction and restored vascularization. a) Immunostaining for microglial marker Iba1 and astrocyte marker GFAP in the mice at 30 days after transplantation. Scale bar, 200 µm. b) Magnification shows the differential immunoreactivity for Iba1 and GFAP in mice with four different treatments. Asterisks indicate ischemic core. Scale bar, 200 µm. c) Immunostaining for glial scar marker CSPG and S100β showing a reduction of glial reaction in mice with cocktail treatment compared with other three groups. Asterisks indicate the ischemic core. Scale bar, 200 µm. d–f) Quantification of the indicated fluorescence intensity (normalized to intact region) in the lesion site 30 days after transplantation. *n* = 5 mice per group. Data are mean ± SEM. ^**^ *p* = 0.0017 in (d), ^*^ *p* = 0.0372 in (e), ^***^ *p* = 0.0004 in (f). g) Quantification of parenchymal volume in the injured area surrounded by GFAP+ cells. *n* = 5 mice per group. Data are mean ± SEM. ^***^ *p* = 0.0009. h) Immunostaining for STEM121 and laminin showing the grafts were vascularized at 30 days after transplantation. Scale bar, 200 µm. i) Quantification of the vascular density in the grafts and intact region. *n* = 5 mice. Data are mean ± SEM. ns *p* = 0.1146. cc, corpus callosum.

In chronic cerebral stroke, glial scar tissues form around the cavity and the cortex often collapses.^[^ ^39^ ^]^ In the mice that received transplantation without the cocktail, the cortex collapsed and few or no GFP+ cells were present at 30 days after transplantation (**Figure** **3a**). Strong glial reaction was present in and surrounding the ischemic site, as evidenced by strong staining for a microglial marker Iba1 and an astrocyte marker GFAP (Figure 3a,b). The Iba1+ microglia/macrophages were present in both the lesion site and surrounding regions, displaying an ameboid morphology (Figure 3b,d). The GFAP+ reactive astrocytes were accumulated at the peri‐infarct to infarct interface (Figure 3b,e), consistent with the robust deposition of chondroitin sulphate proteoglycan (CSPG) around the ischemic site (Figure 3c,f). In contrast, in the mice with the cocktail, the ischemic site was filled with GFP+ cells so that the ischemic core was not collapsed (Figure 3a,g). More importantly, substantially fewer activated microglia and reactive astrocytes were accumulated in and around the ischemic site (Figure 3d,e). The processes of the microglia and astrocytes were thinner (Figure 3b). The expression of CSPG showed a significantly reduced level at the boundary of the cavity (Figure 3c,f), indicating the reduction of glial scar.

In the peri‐infarct area, vascular remodeling contributes to the neuronal survival after ischemic stroke.^[^ ^40^, ^41^ ^]^ Angiogenesis is likely also important for the transplanted cells.^[^ ^20^ ^]^ By immunostaining for laminin, a membrane protein accumulated in blood vessels, we found that blood vessels penetrated into the grafts at 30‐day post transplantation (Figure 3h). Compared to the other four groups in which the vasculature was limited to the edge of the lesion (Figure S3f, Supporting Information), the vascular area in the grafts showed a similar density to that in the intact cortex (Figure 3i), suggesting that grafts fill up the ischemic cavity with vascularization. These results show that successful survival of the graft is accompanied by reduced inflammatory response and diminished glial scar as well as restored vascularization.

### 2.4 NPC Transplantation with the Cocktail Downregulates CCL and CCR5 Expression

**Figure 4:**
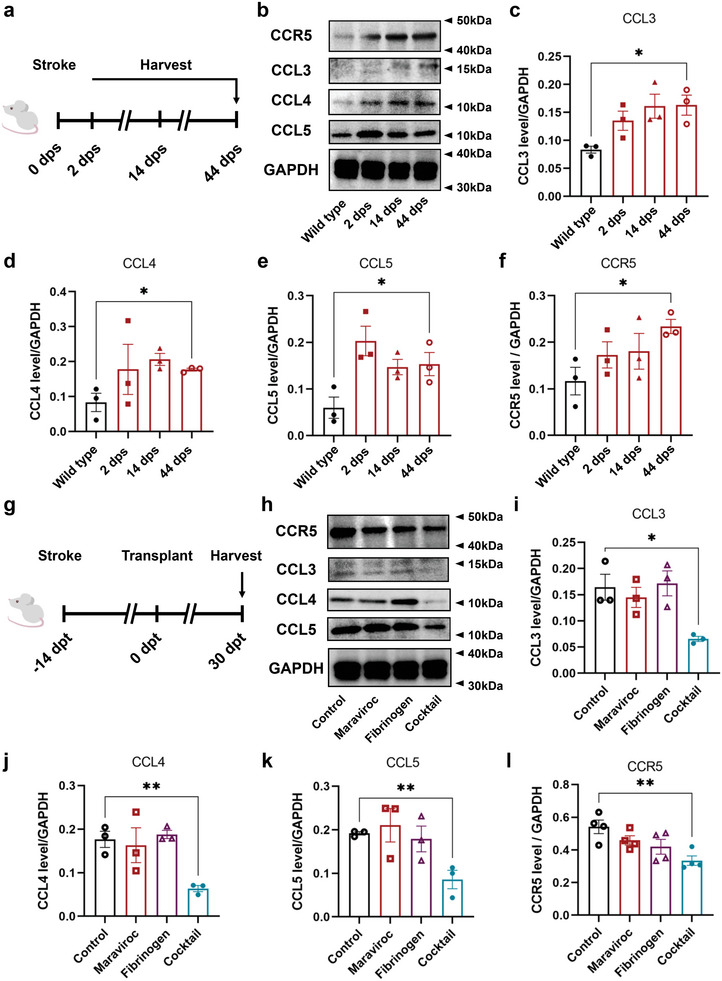
NPC transplantation with the cocktail downregulates CCL and CCR5 expression. a) Experimental strategy for (b–f). b) CCR5, CCL3, CCL4, and CCL5 protein expression in peri‐ and infarct cortex at 2, 14, and 44 days post stroke (dps). c–f) Quantification of CCL3 (c), CCL4 (d), CCL5 (e), and CCR5 (f) protein expression normalized to GAPDH. n = 3 mice per group. Data are mean ± SEM. ^*^ *p* = 0.0136 in (c), ^*^ *p* = 0.0241 in (d), ^*^ *p* = 0.0499 in (e), ^*^ *p* = 0.0244 in (f). g) Experimental strategy for (h–l). h) CCR5, CCL3, CCL4, and CCL5 protein expression in graft and infarct area at 30 days post transplantation. i–l) Quantification of CCL3 (i), CCL4 (j), CCL5 (k), and CCR5 (l) protein expression normalized to GAPDH. *n* = 3 mice per group in (i–k), *n* = 4 mice per group in l. Data are mean ± SEM. ^*^ *p* = 0.0174 in (i), ^**^ *p* = 0.0046 in (j), ^**^ *p* = 0.0079 in (k), ^**^ *p* = 0.0066 in (f).

Ischemic injury results in inflammatory response including the production of cytokines and chemokines. Their receptors, including CCR5, are expressed in mature neurons in the peri‐infarct area after stroke. CCR5 is one of the receptors for chemokine ligands 3 (CCL3), CCL4 and CCL5. CCL3 and CCL4 are two protein components of macrophage inflammatory protein 1 (MIP), also named MIP1‐alpha and beta, respectively. Maraviroc, an antagonist of chemokine receptor CCR5, has been shown to protect the mature neurons bordering the infarct area but not the ischemic core.^[^ ^28^ ^]^ We found that the expression levels of CCR5 and the three ligands were upregulated in the infarct area after stroke, indicated by Western blotting (**Figure** **4**a,b). The increased level of CCR5 and its ligands persisted at 44 days after stroke, suggesting that CCR5 was continuously activated with high concentrations of the ligands in the infarct area during the chronic phase (Figure 4b–f).

The question then is whether maraviroc, fibrinogen, or both modify the expression of CCR5 or CCLs. Western blotting of the transplanted cortical tissues at day 44 after ischemic lesion or 30 days post‐transplantation (Figure 4g) indicated that the CCR5 level was significantly reduced in the cocktail group (Figure 4g). The level of CCL3, 4, 5 did not show obvious difference in the presence of maraviroc or fibrinogen but a substantial reduction in the presence of maraviroc and fibrinogen cocktail (Figure 4g,i,j,k). The results indicate that maraviroc or fibrinogen does not downregulate the CCR5 or CCL3,4,5 levels but the transplantation with the cocktail appears to significantly reduce the presence of CCLs, thus blocking the signaling between CCR5 in injured cortical tissues/grafted NPCs and CCLs produced by inflammatory cells.

### 2.5 Blocking the CCR5 Activation Feedback Mitigates Apoptosis of NPCs

**Figure 5:**
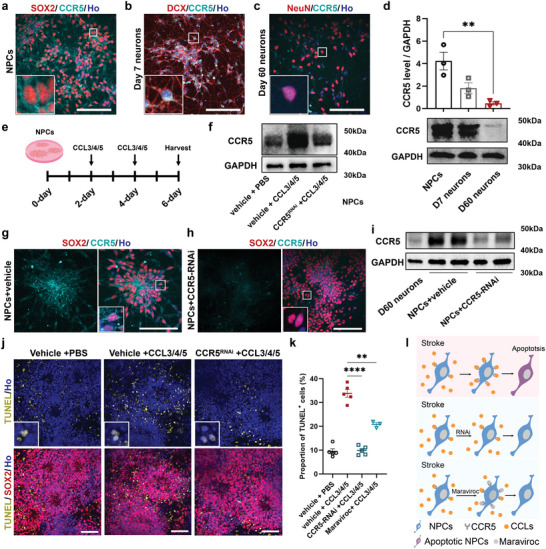
Blocking the CCR5 activation feedback mitigates apoptosis of NPCs. a) Immunostaining for SOX2 and CCR5 showing the high expression of CCR5 on NPCs. Scale bar, 200 µm. b) Immunostaining for DCX and CCR5 showing the high expression of CCR5 on immature neurons. Scale bar, 200 µm. c) Immunostaining for NeuN and CCR5 showing no expression on mature neurons. Scale bar, 200 µm. d) The expression levels of CCR5 on NPCs, immature neurons, and mature neurons. *n* = 3 samples per group. Data are mean ± SEM and relative to GAPDH. ^**^ *p* = 0.0094. e) Experimental strategy for (f–k). f) CCR5 expression levels on NPCs with or without CCR5‐shRNA in the presence or absence CCLs. g) Immunostaining for SOX2 and CCR5 showing the expression of CCR5 on NPCs with vehicle. Scale bar, 200 µm. h) Immunostaining for SOX2 and CCR5 shows that the expression of CCR5 on NPCs was downregulated by CCR5‐shRNA. Scale bar, 200 µm. i) Western blots show the expression levels of CCR5 on the indicated cells. j) Images of NPCs show that CCR5 knockdown reduces the number of apoptotic NPCs (TUNEL+) induced by CCLs. Scale bar, 200 µm. k) Quantification of the percentage of TUNEL+ cells in NPCs. *n* = 5 samples per group. Data are mean ± SEM. ^****^ *p* < 0.0001. l) Schematic showing that blocking the CCR5 activation feedback mitigates apoptosis of NPCs.

CCR5 is upregulated in neurons in the penumbral area after stroke and blocking the CCR5 signaling by genetic means or maraviroc (100 mg k^−1^ g, i.p. daily) promotes the survival of those neurons and their synaptic connections, thus enhancing the behavioral recovery of animals.^[^ ^28^ ^]^ This raises a question of how maraviroc protects the NPCs we transplanted into the ischemic core. Immunostaining for CCR5 along neural differentiation showed that CCR5 is highly expressed on the membrane and cytoplasm of SOX2+ NPCs and DCX+ immature neurons (day 7) but the fluorescent signal is significantly diminished in (day 60) mature neurons (**Figure** **5**a–c). This is confirmed by Western blotting, showing a progressive reduction of its expression (Figure 5d). This result suggests that the NPCs and immature neurons are potentially sensitive for the inflammatory chemokines that are present in and surrounding the ischemic cavity.

We next incubated the NPCs with the three ligands (CCL3, CCL4 and CCL5, 300 ng mL^−1^). Compared with the control group, the proportion of cleaved‐caspase3+ cells was increased in all the three groups (Figure S6a,b, Supporting Information). To mimic the environment in the ischemic infarction, we used the combination of three ligands (100 ng mL^−1^ each of the three chemokines) to incubate the NPCs. Numerous apoptotic and detached NPCs were observed after the treatment (Figure S6b,c, Supporting Information). Interestingly, incubation of the differentiating NPCs with the three ligands from day 2 to 4 induced the upregulation of CCR5 in the cells (Figure 5e,f) and correspondingly increased the proportion of TUNEL+ cells (Figure 5j,k). This result indicates that these chemokines indeed promote the expression of CCR5 on NPCs, inducing apoptosis of NPCs even without microglia.

We then asked if blockade of the chemokine signaling may mitigate the NPC apoptosis. We used lentivirus expressing CCR5‐shRNA to knock down CCR5 expression. As shown by immunostaining and Western blotting, expression of CCR5‐shRNA, but not the control shRNA, significantly reduced the expression of CCR5 in NPCs (Figure 5g–i). Correspondingly, the TUNEL+ cell population was significantly reduced in cultures that were treated with lentivirus carrying CCR5‐shRNA (Figure 5j,k; Figure S7a, Supporting Information). Similarly, blocking CCR5 by maraviroc also reduced the proportion of TUNEL+ NPCs without downregulating the expression of CCR5 (Figure S7a,b, Supporting Information). Thus, the expression of CCR5 on grafted NPCs may be amplified by the inflammatory milieu in the ischemic cavity and blockade of the CCR5 pathway such as by maraviroc may protect the susceptible NPCs from apoptosis (Figure 5l).

## 3 Discussion

We developed a cocktail consisting primarily of maraviroc and fibrinogen to support the survival of grafted NPCs in the ischemic core. In the presence of the cocktail, the human NPCs, transplanted into the ischemic cyst, survive, divide, and then mature, reconstituting the injured cortex in the stroke model by day 30. This is achieved by blocking the signaling between inflammatory chemokines in the ischemic lesion and the high level of CCR5 on NPCs. The survival and maturation of the transplanted NPCs in the ischemic core is accompanied by significant attenuation of glial scar and vascularization of the graft.

Following ischemic stroke, inflammatory cells infiltrate the lesion and release inflammatory cytokines like CCLs. The reactive glial cells form a wall around the lesion site to prevent overflow of pro‐inflammatory mediators. Hence, the inflammatory milieu in the ischemic cavity persists, as we observed. Consequently, NPCs transplanted into the cavity rarely survive and the surviving NPCs, if there are, tend to differentiate to astrocytes.^[^ ^42^ ^]^ It is unclear which inflammatory pathway induces death of the grafted NPCs in the ischemic core. In the penumbra, CCR5 inhibits the expression of PKA and CREB on neurons, leading to an increased loss of dendritic spines and neuronal death. However, this does not explain the low survival of the grafted NPCs in the ischemic core. We found that NPCs express a high level of CCR5, highlighting the sensitivity of NPCs to the inflammatory environment. Its ligands, CCL3/4/5, secreted by infiltrating blood‐born cells upon stroke, activate microglia and astrocytes. Reactive astrocytes and activated microglia also produce CCL3/4/5 along with other cytokines, forming a cascade of inflammatory response. Together, they induce apoptosis of grafted NPCs that express their receptors CCR5. To make it worse, CCLs, present in the inflammatory environment, further stimulate the expression of CCR5. That explains why NPCs, transplanted into the ischemic cavity, rarely survive.^[^ ^16^, ^43^, ^44^ ^]^ Indeed, we observed no survival of the NPCs that were transplanted into the ischemic core. Therefore, experimental cell transplantation therapy mostly targets the healthy brain region adjacent to the ischemic lesion to avoid the toxic environment.^[^ ^8^, ^9^, ^11^, ^45^, ^46^ ^]^ However, transplantation into the penumbra creates an additional injury and poses a substantial risk to patients. Thus, there is a need to develop a way to protect the NPCs that are transplanted into the inflammatory ischemic lesion. The cocktail blocks the CCL‐CCR5 pathway not only on NPCs directly but also on reactive glia, thus protecting the grafted NPCs directly and indirectly through reduced production of CCLs.

Protection of the grafted NPCs has been primarily aimed at neurotrophic support, such as overexpressing SUMO in NPCs, hypoxic treatment, co‐transplantation, and using biomaterials crosslinked with growth factors. These strategies show some efficacy but the neurotrophic support alone is often not sufficient.^[^ ^15^, ^16^, ^38^, ^47^ ^]^ Since inflammation is the leading cause of cell death, blocking the inflammatory signaling may be necessary to prevent the cells from death. Indeed, we found that blocking the CCL‐CCR5 signaling in NPCs either by a genetic means (RNAi) or a chemical antagonist of CCR5 maraviroc, even in the in vitro system, is sufficient to prevent apoptosis of NPCs. A recent study showed that administration of maraviroc (100 mg k^−1^ g, i.p. daily) rescues the neurons from death in the peri‐infarct region.^[^ ^28^ ^]^ Since there is a lack of blood flow in the ischemic cavity, peripheral administration of medications is unlikely to affect the NPCs grafted in the cyst. We hence transplanted the NPCs in the presence of maraviroc but that is not sufficient to rescue any grafted cells. We reasoned that it is possibly due to the rapid dilution and/or degradation of maraviroc in vivo. Indeed, when we combined maraviroc with fibrinogen, which forms a degradable gel in the presence of thrombin or Ca++ and which slows the release of maraviroc, the grafted NPCs survive, even though fibrinogen itself did not support the survival of transplanted NPCs. Since fibrinogen is neurotrophic and it is gellable, the fact that it does not support the survival of NPCs suggests that a simple holding of grafted cells in the cyst and neurotrophic is not sufficient for cell survival. Mitigating the inflammatory insult in such an environment may be necessary.

The surviving human NPCs appear to proliferate and fill the entire cyst within 30 days post‐transplant. Strikingly, the glial scar surrounding the ischemic cavity is greatly reduced, as evidenced by the substantial reduction of GFAP and IBA1 immunoreactivity and re‐vascularization of the graft, indicated by laminin‐labeled blood vessels. Such a tissue remodeling is likely the outcome of the complex interactions between the transplanted cells and host cells besides the effect maraviroc.^[^ ^48^, ^49^, ^50^, ^51^ ^]^ Perhaps most strikingly, the vast majority of the transplanted NPCs become post‐mitotic neurons within 30 days, indicated by their expression of NeuN and NF. Human cortical NPCs tend to proliferate for a long period before differentiating into mature neurons,^[^ ^45^, ^52^, ^53^ ^]^ which explains the proliferation of surviving NPCs and filling of the cyst within 30 days. The rapid differentiation/maturation may be due to the lack of growth factors in the environment. In the presence of growth factors, spinal NPCs, transplanted into the cyst of injured spinal cord, retained the progenitor state for several months.^[^ ^6^ ^]^ Hence, the cocktail we described here offers a base medium for safe cell transplantation therapy. It may be supplemented with growth factors like FGF2 to promote proliferation, or neurotrophic factors like BNDF and GDNF for further survival and maturation, or notch inhibitors like compound E to enhance cell cycle exit depending on the number and developmental stage of the donor cells and the size of the lesion to repair. While the base cocktail may be modified to fit the need depending on the nature of the disease and the properties of the NPCs, our cocktail opens the possibility to enable survival of the transplanted NPCs and repair the gap lesions like stroke and other inflammatory neurological conditions through cell transplantation therapy. The present study focuses on cell survival following NPC transplantation at the chronic stage. The survival, migration, and maturation may be different when transplanted at the acute or subacute stages. Furthermore, while we have shown the survival and differentiation of transplanted NPCs as well as graft vascularization at one‐month post‐transplantation, longer‐term integration and functional contribution is needed, which is our ongoing effort.

## 4 Experimental Section

All experiments were carried out according to methods approved by the Institutional Animal Care and Use Committees at Duke‐NUS Medical School (protocol 2018/SHS/1410).

### Cell Culture

Human embryonic stem cells (ESCs, H9 and eGFP‐H9 hESC lines) were cultured in a feeder‐free mTeSR medium with 1 × mTeSR supplement, 1 × Non‐Essential Amino Acids (NEAA) and 1 × Glutamax in a Matrigel‐coated 6‐well plate. ESCs were fed daily and passaged twice a week.

Induction of forebrain glutamate neurons was described.^[^ ^54^ ^]^ Briefly, H9 hESCs or eGFP H9 hESCs were cultured on 6‐well plates coated with Matrigel/vitronectin for one week. ESC colonies were gently blown off by 1 mL pipettes to form cell aggregates at day 0. Cell aggregates were cultured in flasks for 7 days with the neural induction medium (NIM) consisting of DMEM/F12, 1 × N2 supplement, 1 × NEAA, 2‐µM SB431542, and 2‐µM DMH‐1. Cell aggregates were adhered to 6‐well plates in the presence of NIM with 5% FBS for 6 h, and then replaced with fresh NIM. The aggregates were fed with NIM till neural rosette formation at day 16. The rosettes were gently blown off using 1 mL pipettes and suspended in flasks with NIM for 7 days. Then, NIM was changed every four days from day 23. The neural progenitors were maintained in NIM till transplantation or immunostaining. Progenitors were digested into single cells using TrypLE for 3 min at day 49. After one additional day of culture in NIM supplemented with 1 × B27 and 100 nm compound E, the progenitors were collected for transplantation in ischemic stroke models. For immunostaining, progenitors were seeded on glass coverslips and staining was performed after one‐week culture.

To test the effects of CCR5 activation on progenitors and neurons, cells were seeded on Matrigel coated coverslips or 6‐well plates and treated with overdose (300 ng mL^−1^) ligands of CCR5. CCL3, CCL4, CCL5 and their combination (CCL3/4/5) were respectively added into NIM every other day. After four‐day treatment, progenitors or neurons were collected for staining or immunoblotting.

### Lentivirus Production

Human CCR5 29mer shRNA plasmids (pRS_hU6_CCR5shRNA_SV40_Puro) and non‐effective 29‐mer scrambled shRNA cassette in pRS Vector were obtained from OriGene (CAT#: TR314126, CAT#: TR30012). The lentiviral shRNAs were generated in the HEK 293FT cell line by transfecting with the packaging and backbone plasmids. HEK 293FT cells were cultured in DMEM with 10% FBS. The supernatant was collected after 3‐day culture. Viral particles were concentrated by ultracentrifugation at 25 000 rpm for 2.5 h at 4 °C. The viral particles were resuspended in DMEM.

### Transduction of shRNA

One hundred thusand NPCs were seeded on coverslips in each well of a 24‐well plate for two days to reach 50% confluency upon transduction at 37 °C in a humidified 5% CO_2_ incubator. The lentiviral shRNAs (MOIs of 20) were used to infect NPCs overnight at 37 °C. The medium containing lentiviral particles was removed from wells and replaced with 500 µL fresh pre‐warmed NIM. The infected NPCs were collected for immunostaining or western blotting at 5 days after transduction.

### Preparation of the Cocktail

Stock solution of 30 mg mL^−1^ fibrinogen, 50 mg mL^−1^ maraviroc and 250 mM CaCl_2_ (100×) were prepared in the following manner: fibrinogen (F3879, Sigma) was dissolved in a‐CSF for 1 h at room temperature. Maraviroc was dissolved in Dimethyl Sulfoxide (DMSO). CaCl2 (Sigma) was dissolved in deionized (DI) water. All solutions were sterile filtered and stored at −20 °C for use. Fibrinogen stock solution was diluted to 10 mg mL^−1^ fibrinogen in a‐CSF before preparing the cocktail. Cocktail was made of 50 mg mL^−1^ maraviroc and 10 mg mL^−1^ fibrinogen in a volume ratio of 1: 9 with 2.5 mm CaCl_2_.

### Release Profile of Maraviroc within Cocktail Gel

The wavelength of maximum absorbance (*λ*_max_) of maraviroc in Phosphate buffer (pH 7.4) was found 210 nm by scanning them over the UV range of 2000 to 400 nm. Standard drug solution of maraviroc was prepared by dissolving 50 mg pure maraviroc in phosphate buffer (pH 7.4) and transferred into 5 ml volumetric flask to obtain 10 mg ml^−1^ of stock solution and the resulting maraviroc. Solution was used as working standard solution from which desired concentrations of solution were prepared. The final concentration of 0, 0.5, 1.0, 1.5, 2.0, 2.5, 3.0, 3.5, 4.0, 4.5, 5.0, 5.5, 6.0 mg ml^−1^ and absorbances were taken at *λ*_max_ 210 nm using an appropriate blank. Inducing the gelation of 200ul fibrinogen (9 mg ml^−1^) by thrombin(50) in vitro, and the gel with 1 mg maraviroc was incubated in 1 ml PBS with pH 7.4 at 37 °C. For free drug group, 5 mg maraviroc was directly dissolved in 1 ml PBS. Fifty microliters of solution were taken to test the absorbance by Microplate Absorbance Spectrophotometer (Bio‐RAD, xMark). The final concentrations of maraviroc in PBS were caculated according to calibration curve.

### Stroke Model and Cell Transplantation

All animal studies were performed in accordance with the institutional animal care and use committee at Duke‐NUS Medical School. Ischemic strokes in adult (10–12 weeks) male SCID mice were induced through photo‐thrombosis. Briefly, Rose Bengal was administered intravenously at a dose of 0.1 mg g^−1^ per mouse. Mouse skull was exposed under 2% isoflurane anesthesia. The right motor cortex (anterior‐posterior [AP] = +2 mm, lateral [L] = +1 mm) received a 2.5‐mm diameter illumination of cold light through the intact skull for 15 min. Animals were randomly grouped and transplanted with forebrain glutamatergic progenitors. Fifty thousand cells were resuspended in 1 µl artificial cerebral spinal fluid (a‐CSF) in the presence or absence of maraviroc (5 mg mL^−1^) or fibrinogen (10 mg mL^−1^) and injected into the injured site ([AP] = +2 mm, [L] = +1 mm, vertical [V] = −1.5 mm, from dura).

### Tissue Preparation and Immunohistochemistry

Animals were sacrificed with a lethal dose of pentobarbital (250 mg kg^−1^) and immediately perfused with PBS followed by 4% cold paraformaldehyde (PFA). The brain samples were fixed in cold PFA for 2 h and immersed in sequentially in 20% and 30% sucrose at 4 °C until sunk. Serial coronal (1.54 to ‐0.22 mm from Bregma) sections were collected on a freezing microtome at a 40‐m thickness and stored at −20 °C. For immunostaining, sections were incubated with blocking solution containing 10% normal donkey serum and 0.2% TritonX‐100 for 1h at room temperature. Then sections were incubated with primary antibodies overnight at 4 °C (**Table** **1**).

**Table 1:** Primary Antibodies.

Antibodies	SOURCE	IDENTIFIER	
anti‐GFAP	Cell Signaling Tech	34001	https://www.cellsignal.com/products/primary‐antibodies/gfap‐e6n9l‐mouse‐mab/34001?N=4294956287amp;Ntt=gfap&Nrpp=60&No=%7Boffset%7D&fromPage=plp
anti‐GFAP	Agilent Dako	Z0334	https://www.agilent.com/en/product/immunohistochemistry/antibodies‐controls/primary‐antibodies/glial‐fibrillary‐acidic‐protein‐(concentrate)‐76683
anti‐SOX2	R&D Systems	AF2018‐SP	https://www.rndsystems.com/products/human‐mouse‐rat‐sox2‐antibody‐245610_mab2018
anti‐DCX	Cell Signaling Tech	4604	https://www.cellsignal.com/products/primary‐antibodies/doublecortin‐antibody/4604
anti‐Cleaved‐caspase3	Cell Signaling Tech	9661S	https://www.cellsignal.com/products/primary‐antibodies/cleaved‐caspase‐3‐asp175‐antibody/9661
anti‐STEM121	Takara	Y40410	https://www.takarabio.com/products/antibodies‐and‐elisa/primary‐antibodies‐and‐elisas‐by‐research‐area/stem‐cell‐research‐antibodies/stem‐antibodies?catalog=Y40410
anti‐NF‐L	Abcam	ab7255	https://www.abcam.com/68kda‐neurofilamentnf‐l‐antibody‐da2‐ab7255.html
anti‐NeuN	Merck Millipore	MAB377	https://www.merckmillipore.com/CN/zh/product/Anti‐NeuN‐Antibody‐clone‐A60,MM_NF‐MAB377
anti‐CCR5	Abcam	ab110103	https://www.abcam.com/ccr5‐antibody‐t218‐ab110103.html
anti‐Ctip2	Abcam	ab18465	https://www.abcam.com/ctip2‐antibody‐25b6‐ab18465.html
anti‐Brn2	Proteintech	14596‐1‐AP	https://www.ptgcn.com/products/POU3F2‐Antibody‐14596‐1‐AP.htm
anti‐Foxp2	Abcam	ab16046	https://www.abcam.com/foxp2‐antibody‐ab16046.html
anti‐Ki67	Cell Signaling Tech	9129S	https://www.cellsignal.com/products/primary‐antibodies/ki‐67‐d3b5‐rabbit‐mab/9129
anti‐Sox9	R&D Systems	AF3075	https://www.rndsystems.com/products/human‐sox9‐antibody_af3075
anti‐S100β	Abcam	ab52642	https://www.abcam.com/s100‐beta‐antibodyep1576y‐astrocyte‐marker‐ab52642.html
anti‐CSPG	Abcam	ab11570	https://www.abcam.com/chondroitin‐sulfate‐antibody‐cs‐56‐ab11570.html
anti‐Iba1	Wako Pure Chemical Corporation	019‐19741	https://labchem‐wako.fujifilm.com/us/product/detail/W01W0101‐1974.html
anti‐Laminin	Novusbio	NB300‐144	https://www.novusbio.com/products/laminin‐antibody_nb300‐144
anti‐MAP2	Cell Signaling Tech	8707S	https://www.cellsignal.com/products/primary‐antibodies/map2‐d5g1‐xp‐rabbit‐mab/8707?applicationCode=IF&productId=3658&Ns=product.status.releasedDateIso%7C1&N=4294967218+4294956287&Nrpp=200&No=2600&fromPage=plp

Sections were subsequently rinsed with PBS and incubated with corresponding secondary antibodies for 1 h at room temperature. Immunolabeled sections were mounted by Fluoromount‐G with Hoechst. For TUNEL staining, cells on coverslips were fixed in 4% PFA for 30 min, and then incubated with Terminal Deoxynucleotidyl Transferase (TdT) Equilibration buffer (Elabscience) at 37 °C for 30 min. Progenitors were incubated in Labeling solution (Elabscience) with TdT enzyme at 37 °C for 1 h.

### Imaging and Cellular Quantification

To quantify the population of DCX, SOX2, Ki67, NF and NeuN positive cells among total grafted cells (eGFP and Hoechst co‐labeled), one brain slice was selected from every 6 serial slices for stereological counting on a Zeiss M1 Microscope with Stereo Investigator software (MBF Bioscience). In brief, immunolabeled slices were scanned on Zeiss M1 Microscope, grafts area was outlined manually, and then corresponding fluorescence labeled cells were unbiasedly counted. Population of cleaved‐caspase3 expressing cells among total grafted cells was counted with the ImageJ software. Data were replicated 4 to 6 times per group. To quantify the population of Brn2, Ctip2 and Foxp2 expressing cells among total GFP+ cells on coverslips, all coverslips were scanned and captured by 20× objective with a confocal microscope (Nikon), and then was counted with the ImageJ software. Data were replicated three times. All data were expressed as means±SEM.

### Infarct Area Analysis and Quantification

The infarct area was defined by GFAP, S100β, CSPG and Iba1 staining. Fluorescence intensity in the infarct area was measured with ImageJ software and normalized to the surrounding intact region. To measure the thickness of infarct area, six slices were chosen randomly from 35 brain slices and captured by a confocal microscope (Nikon). The vertical distance from surface in epicenter to corpus callosum was measured with ImageJ software. All data were replicated four to six times and were expressed as means±SEM.

### Behavior Tests

Mice (*n* = 9–10 per group) were tested on the rotarod and grid‐walk tasks. Behavior was assessed −14, 0, 14 and 30 days following transplantation. For the rotarod test, latency of fall was calculated to assess the motor function. For the grid‐walk test, deficit was calculated as the number of impaired limb (right foot) within 10 min.

### Isolation of Injured Tissue

Animals were anaesthetized and perfused with cold PBS at 2, 14, and 44 days after stroke. The injured cortices or grafts (an area with a radius of 1 mm from the epicenter) were dissected manually under a stereomicroscope (Zeiss) and stored at −80 °C.

### Western Blotting

Progenitors and neurons were rinsed using PBS and resuspended in RIPA buffer with protease inhibitor and phosphatase inhibitor. Samples were collected to 1.5 mL tubes on ice for 15 min. For tissue samples, extracts were sonicated in cold RIPA buffer with protease/phosphatase inhibitors. All samples were quantified using Quick Start Bradford Protein Assay (Bio‐RAD), and then Laemmli buffer (Bio‐RAD) was added to each tube. Samples were heated at 95 °C for 5 min and stored at −80 °C. Total 15 µg of extract was loaded to each well on a 10% Bis‐Tris pre‐casted gel for electrophoresis.

After running at 120 V for 40 min, proteins were transferred to PVDF membranes at 400 mA for 30 min. Membranes were subsequently blocked in 0.1% TBS‐Tween (TBST) with 5% non‐fat milk for 1 hour. Then the membranes were incubated with primary antibodies overnight at 4 °C: anti‐CCR5 (1:1000, Abcam, ab110103), anti‐CCL3 (1:1000, Abcam, ab259372), anti‐CCL4 (1:1000, Abcam, EP521Y), anti‐CCL5 (1:1000, Thermo Fisher Scientific, 701 030), anti‐GAPDH (1:5000, Thermo Fisher Scientific, MA5‐15738). The membranes were washed three times by 0.1% TBST and incubated with corresponding secondary antibodies at room temperature for 1 h. The protein bands were presented by Enhanced Chemiluminescence Substrate (Promega) and visualized in Bio‐RAD Chemidoc system. All intensity of bands was analyzed by ImageJ software and normalized to corresponding GAPDH bands.

### Quantification and Statistical Analysis

The data of the stroke group and the transplanted groups are normally distributed (the mean ± SD is listed in Table S1, Supporting Information). Unpaired *t*‐test was used for comparison between two groups. Error bars in all figures represent means ± SEM. Differences were considered statistically significant at a *p*‐value of <0.05. All data were analyzed using GraphPad Prism.

## Conflict of Interest

The authors declare no conflict of interest.

## Author Contributions

S.‐C.Z. and Z.W. conceptualized and designed the experiments; Z.W. performed cell transplantation; Z.W., D.Z., Y.S.T., and F.Y. performed cell culture; Q.Y. and Z.W. bred the animals; Y.S.T. and Z.W. performed lentivirus production; Z.W. and D.Z. performed data collection; Z.W., D.Z., and S.‐C.Z. performed data analysis and interpretation; Z.W. and S.‐C.Z. drafted the article.

## Supporting information

Supporting Information

Supporting Information

## Data Availability

The data that support the findings of this study are available on request from the corresponding author. The data are not publicly available due to privacy or ethical restrictions.
