# Supplementary material for: Enabling Survival of Transplanted Neural Precursor Cells in the Ischemic Brain
Source: Adv Sci (Weinh). 2023 Oct 22;10(33):2302527. doi: 10.1002/advs.202302527 (PMC10667812; doi:10.1002/advs.202302527)
Supplement: Supplementary file 1 — Supporting Information [file ADVS-10-2302527-s001.pdf]

ADVANCED SCIENCE
Open Access

Supporting Information

for *Adv. Sci.*, DOI 10.1002/advs.202302527

Enabling Survival of Transplanted Neural Precursor Cells in the Ischemic Brain

*Zhifu Wang, Danyi Zheng, Ye Sing Tan, Qiang Yuan, Fang Yuan and Su-Chun Zhang**

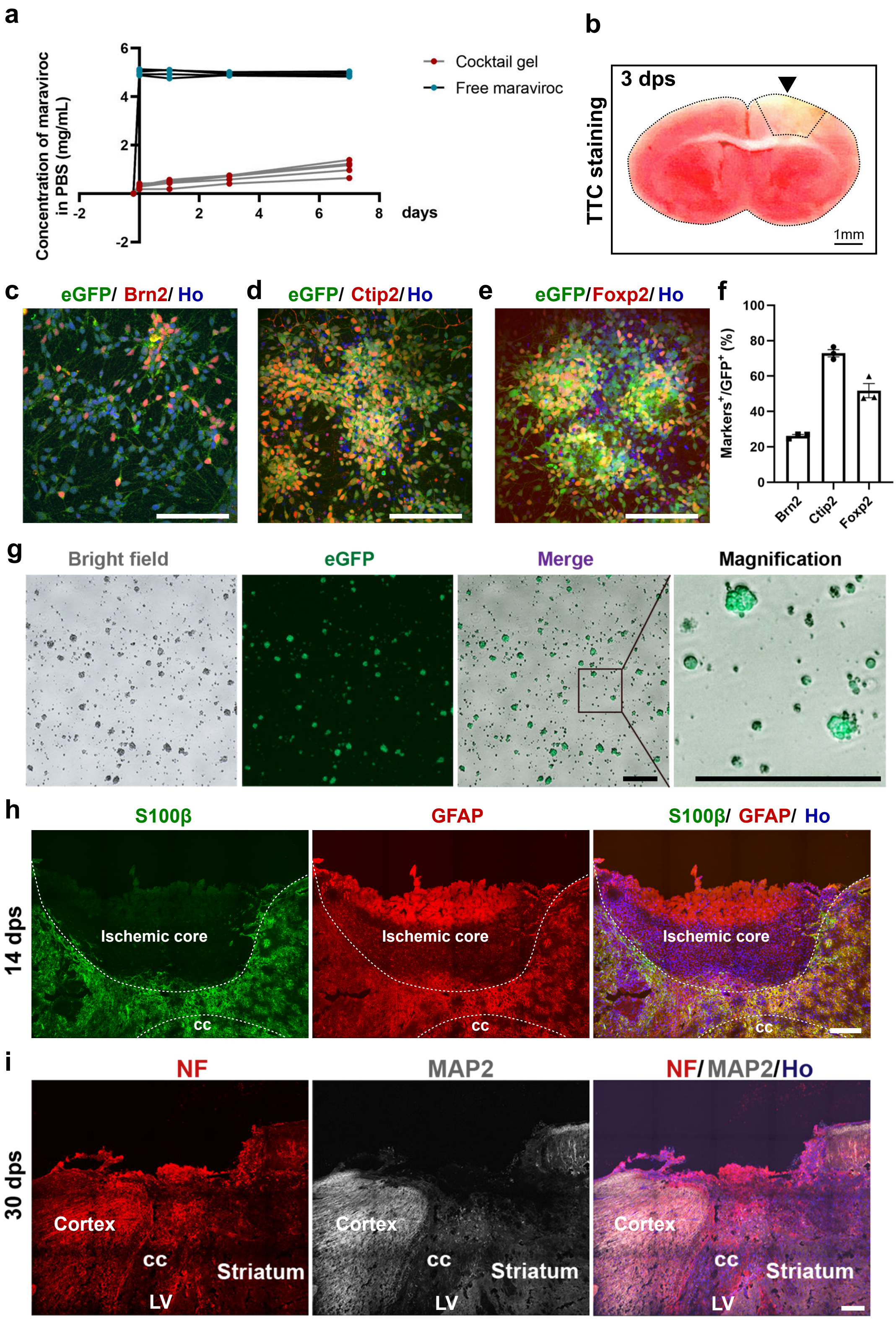

Supplementary Figure.1 Construction of photothrombosis induced ischemic stroke and preparation of NPCs for transplantation.

a, *In vitro* maraviroc release profile of the cocktail gel and free drug. **b**, Triphenyl tetrazolium chloride (TTC) staining on brain slices showing the infarct area at 3 days post stroke (dps). Black arrowheads indicate the infarct area. Scale bar, 1 mm. **c-e**, Immunostaining for cortical markers Brn2 (upper layer, **c**), Ctip2 (deep layer, **d**), and Foxp2 (deep layer, **e**) shows the different subtypes of cortical progenitors for transplantation. Scale bar, 200 μm. **f**, Quantification of the percentage of indicated NPCs in GFP+ cells. n=3 samples. Data are mean ± SEM. **g**, Images of dissociated GFP+ NPCs before transplantation. Scale bar, 200 μm. **h**, Immunostaining for astrocyte markers GFAP and S100β showing the ischemic core is surrounded by reactive astrocytes at 14-dps. Dotted lines indicate the ischemic core and corpus callosum (cc). Scale bar, 200 μm. **i**, Immunostaining for neurite markers NF and Microtubule-associated protein 2 (MAP2) on the cortical sections shows the injured cortex has been collapsed without neurites at 30-dps. Scale bar, 200 μm.

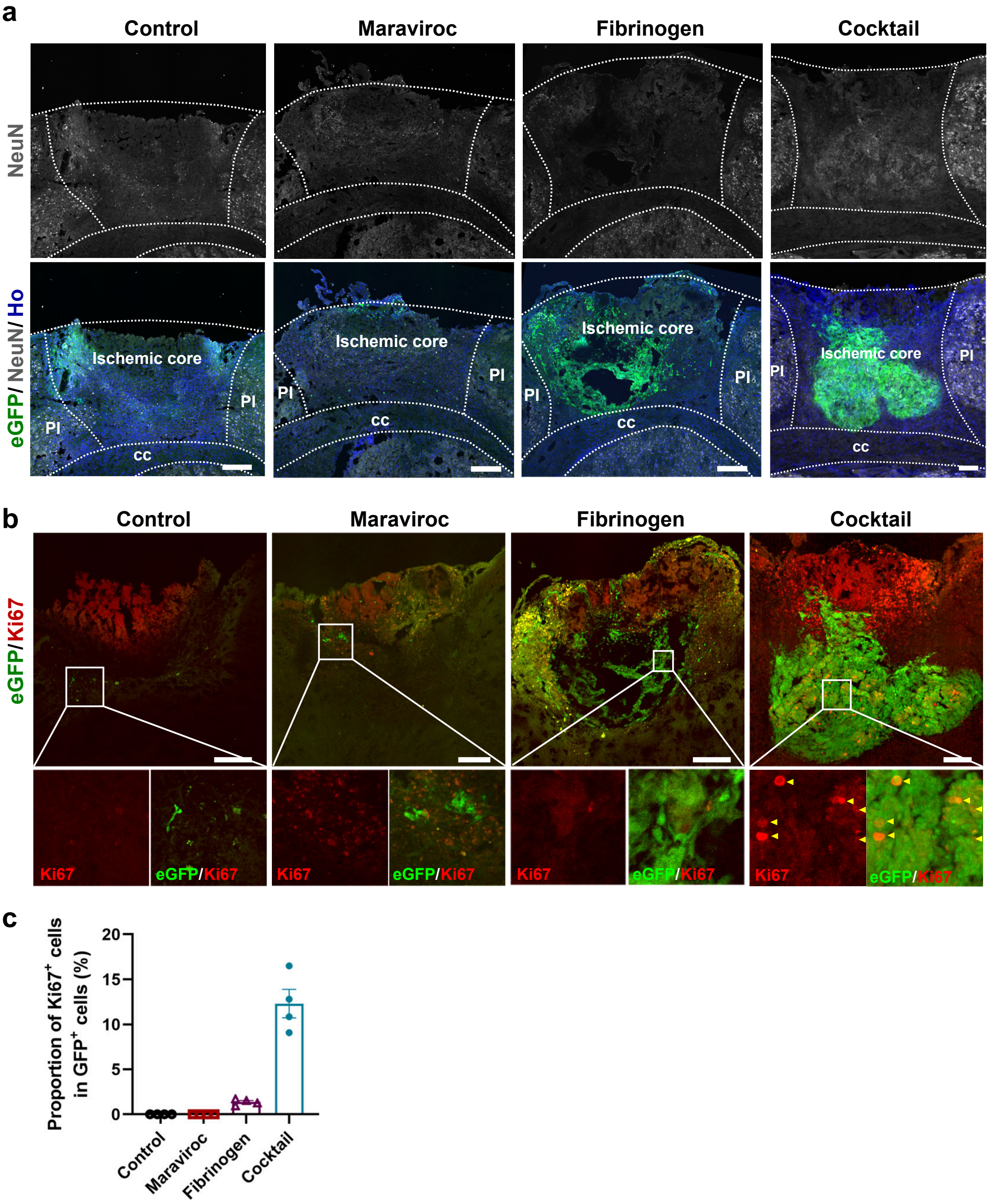

Supplementary Figure.2 The grafted NPCs with cocktail enable to proliferate in the ischemic core at 7 days after transplantation.

a, Immunostaining for NeuN shows that the NPCs (GFP+) were transplanted in the ischemic core (NeuN-) at 7 days after transplantation. Scale bar, 200 μm. **b**, Immunostaining for proliferation marker Ki67 shows the expression of Ki67 on grafted cells at 7 days after transplantation. The boxed areas are magnified in the bottom panel. Yellow arrowheads indicate Ki67 and GFP co-labeled cells. Scale bar, 200 μm. **c**, Quantification of the percentage of Ki67+ cells in grafted cells (GFP+), showing over 10% of grafted cells express proliferation marker Ki67 in the cocktail group. n=4 mice per group. Data are mean±SEM. PI, peri-infarct. cc, corpus callosum.

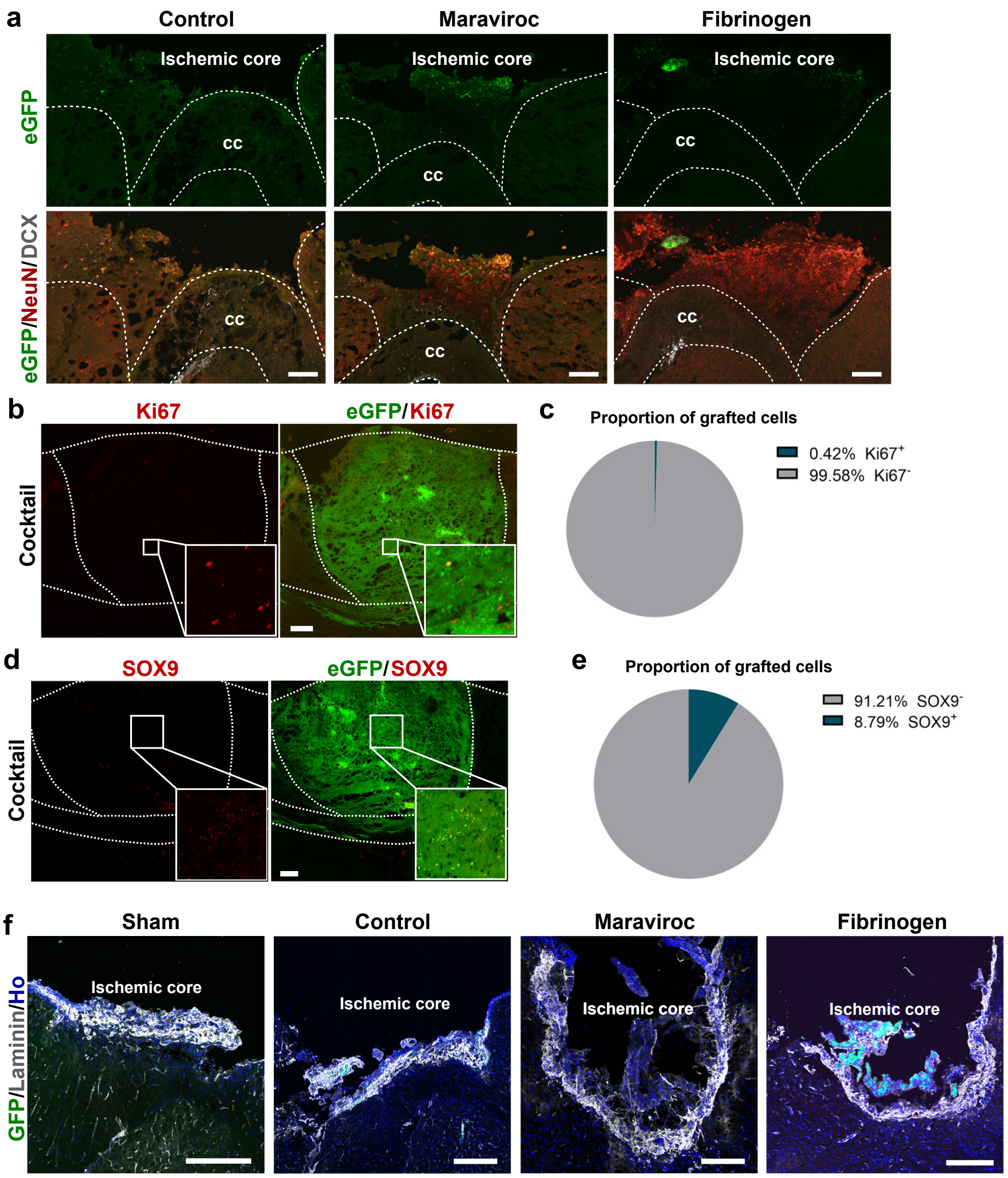

Supplementary Figure.3 Grafted cells with the cocktail treatment survive in ischemic core at 30 days after transplantation.

a, Immunostaining for NeuN and DCX in the mice transplanted with NPCs (GFP+) in the indicated medium, showing no GFP+ cells survive without cocktail in the ischemic core. Scale bar, 200 μm. **b**, Images of the grafts show the expression of Ki67 on grafted cells (GFP+) at 30 days after transplantation. Scale bar, 1 mm. **c**, Quantification of the percentage of Ki67+ cells in the grafted cells indicates that few grafted cells enable to proliferate at 30 days after transplantation. n=4 mice. **d**, Immunostaining for astrocyte marker SOX9 in the mice transplanted with NPCs in the cocktail at 30 days after transplantation. Scale bar, 1 mm. **e**, Quantification of the percentage of SOX9+ cells in the grafted cells shows that about 9% of the grafted cells are astrocytes. n=4 mice. **f**, Immunostaining for GFP and laminin showing the collapsed cortex of four groups at one month post transplantation. Scale bar, 500 μm. cc, corpus callosum.

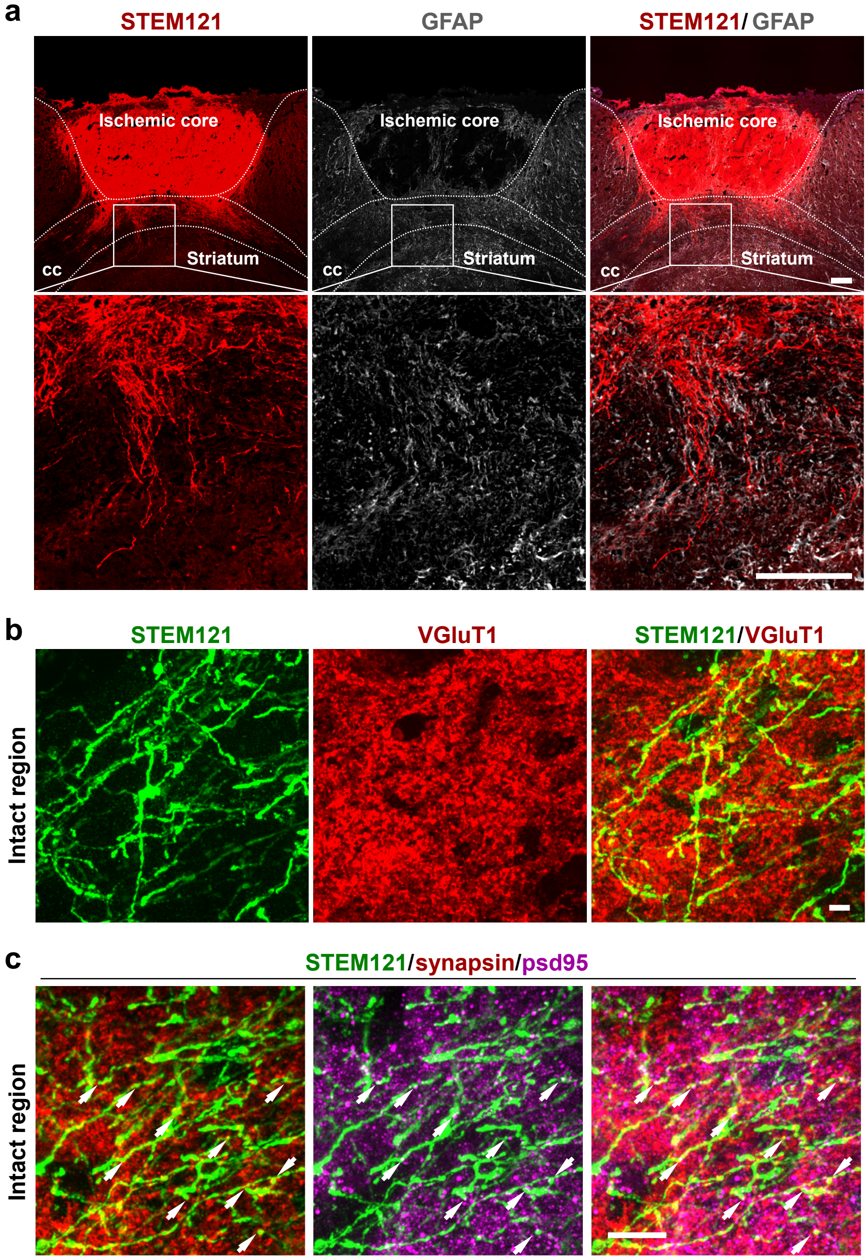

Supplementary Figure.4 Grafted cells with the cocktail treatment project axons out of the ischemic core at 30 days after transplantation.

a, Immunostaining for STEM121 and GFAP in the mice transplanted with cocktail and NPCs at 30 days after transplantation, showing that axons grew out through the glial scar. Scale bar, 200 μm. **b**, Immunostaining for STEM121 and glutamatergic marker VGluT1 showing that grafted cells differentiated to glutamatergic neurons. Scale bar, 10 μm. **c**, Immunostaining for STEM121, synapsin(pre-synaptic marker), and psd95 (post-synaptic marker) at the undamaged region adjacent to injured site, showing that neurites of grafted neurons form synapses (white arrows) with host neurons. Scale bar, 10 μm. cc, corpus callosum.

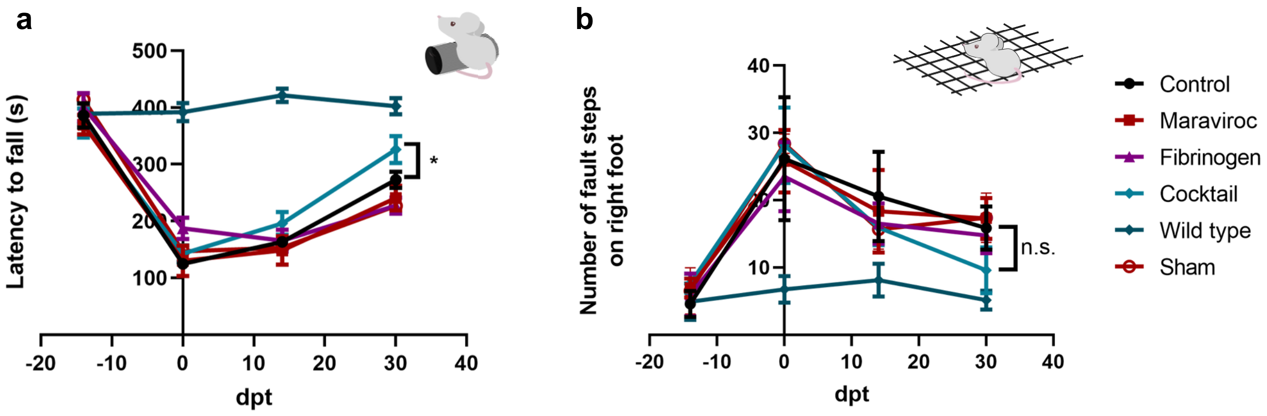

Supplementary Fig. 5. Behavioral tests.
Behavioral tests were performed at -14 days (pre-stroke), 0 days, 14 days, and 30 days post transplantation. **a**, Quantification of the latency to fall on the rotarod test showing the motor recovery at 30 days post transplantation. n=9 mice for maraviroc and fibrinogen groups, n=10 mice for all other groups. Data are mean ± SEM. * p=0.034 (cocktail vs control). **b**, Data on motor performances assessed with grid-walking tests. n= 9 mice for all groups in **b**. Data are mean ± SEM. n.s. p=0.053 (cocktail vs control).

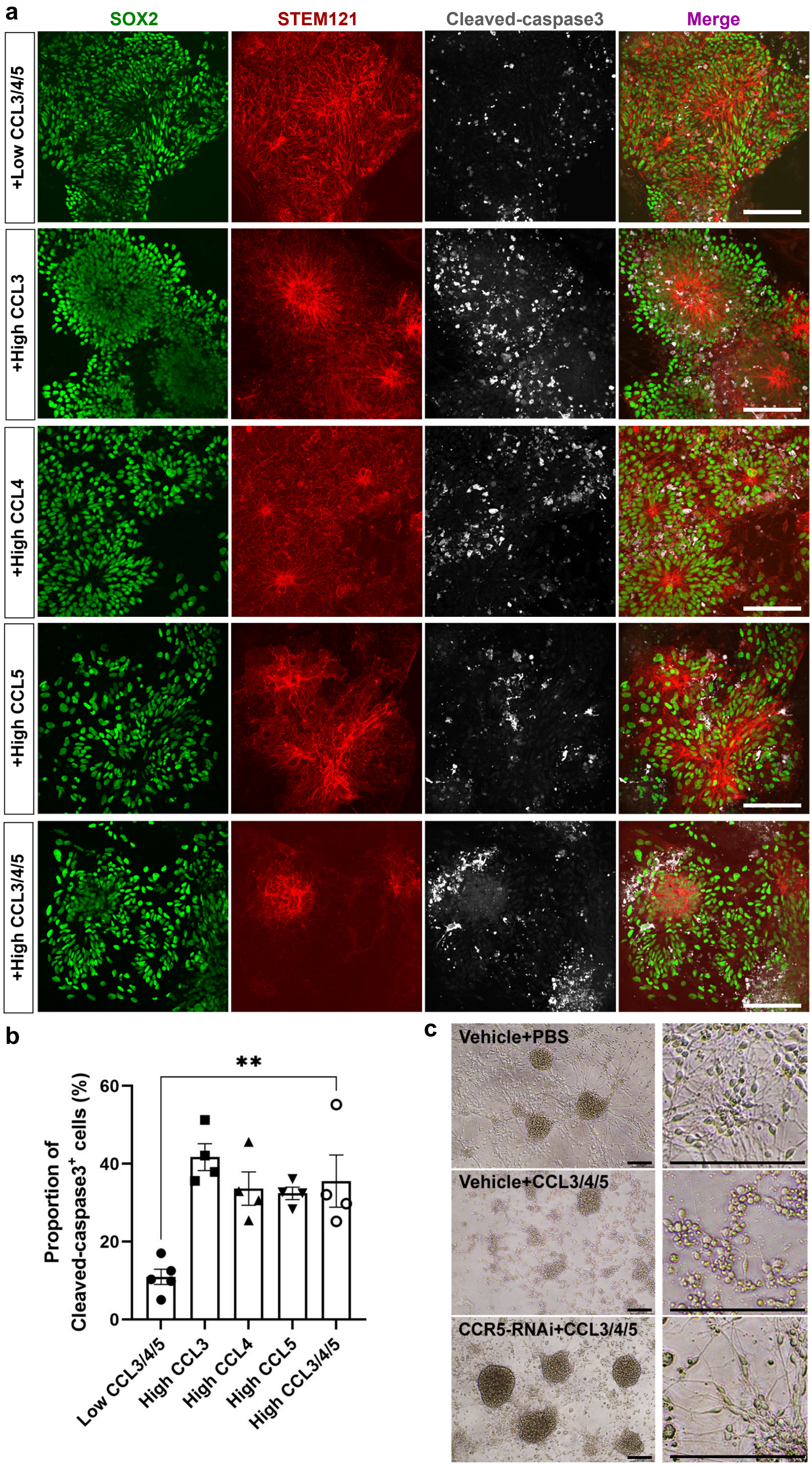

Supplementary Figure.6 High concentrations of CCLs induce the apoptosis of NPCs.

a, Immunostaining for SOX2, STEM121, and apoptotic marker cleaved-caspase3 shows the differential expression of cleaved-caspase3 on the NPCs with the indicated treatment. Scale bar, 200 μm. **b**, Quantification of the percentage of cleaved-caspase3+ cells shows that the high concentration (300 ng/mL) of CCLs induce significant apoptosis versus the low concentration (10 ng/mL). n=5 samples for low CCLs group, n=4 samples for other four groups. Data are mean±SEM. **p=0.0046 **c**, Images of the NPCs and immature neurons with the indicated treatment. Scale bar, 200 μm.

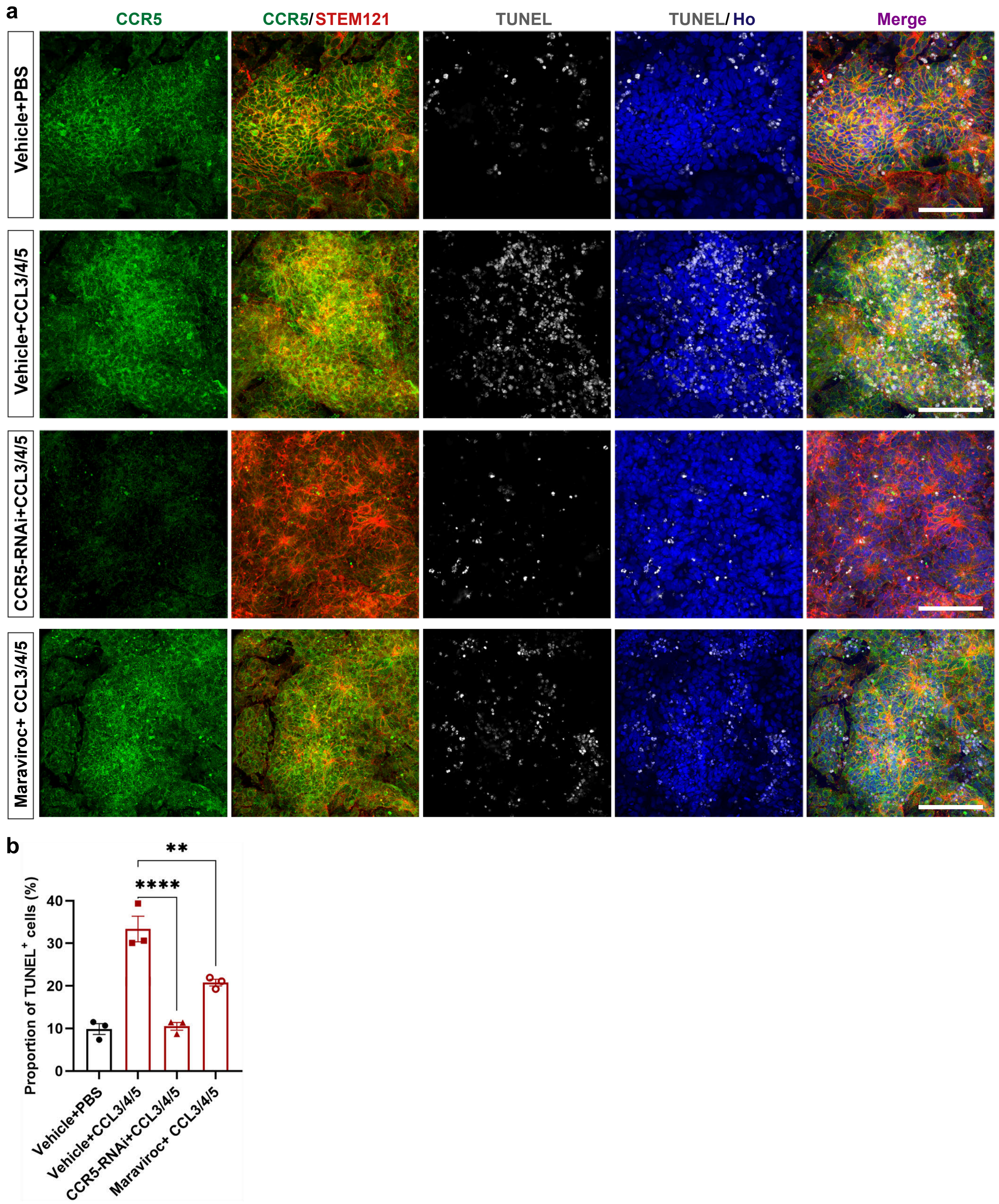

Supplementary Figure.7 Blocking the CCR5 activation feedback mitigates apoptosis of NPCs.

a, Fluorescent staining for CCR5, STEM121, and TUNEL in the NPCs with the indicated treatment. Scale bar, 200 μm. **b**, Quantification of the proportion of the apoptotic cells (TUNEL+) in NPCs shows that blocking the CCR5 activation by shRNA or small molecule (maraviroc) reduces the apoptosis of the NPCs. n=3 samples per group. Data are mean ± SEM. **p=0.0041, ****p<0.0001.

				Mean	SD
Fig 1	1e	Control	cleaved-caspase3+	97.72	1.55
			cleaved-caspase3-	2.28	
		Maraviroc	cleaved-caspase3+	93.92	2.07
			cleaved-caspase3-	6.08	
		Fibrinogen	cleaved-caspase3+	89.18	4.48
			cleaved-caspase3-	10.82	
		Cocktail	cleaved-caspase3+	0.66	0.39
			cleaved-caspase3-	99.34	
	1g	Control	DCX+	31.60	14.24
			SOX2+	52.00	24.65
		Maraviroc	DCX+	36.00	18.48
			SOX2+	49.25	25.10
		Fibrinogen	DCX+	388.25	196.13
			SOX2+	731.25	178.37
		Cocktail	DCX+	96505.80	13283.02
			SOX2+	20760.00	6723.71
Fig 2	2g	Cocktail	NF+	89.175	5.388124
			NF-	10.825	
	2i	Cocktail	NeuN- STEM121+	1045610.46	63403.45
			NeuN+ STEM121+	740129.56	144323.50
	2m	Cocktail	Upper layer	70.97	7.21
			Deep layer	43.72	4.27
Fig 3	3d	Sham	Iba1	8.23	2.09
		Control	Iba1	7.50	1.62
		Maraviroc	Iba1	7.68	1.78
		Fibrinogen	Iba1	5.95	2.09
		Cocktail	Iba1	1.48	0.44
	3e	Sham	GFAP	3.88	1.10
		Control	GFAP	3.50	0.46
		Maraviroc	GFAP	3.20	0.70
		Fibrinogen	GFAP	3.70	0.67
		Cocktail	GFAP	1.80	0.36
	3f	Sham	CSPG	5.20	0.68
		Control	CSPG	5.43	0.86
		Maraviroc	CSPG	4.58	1.47
		Fibrinogen	CSPG	4.88	1.66
		Cocktail	CSPG	0.33	0.15
	3g	Sham		0.17	0.12
		Control		0.14	0.09
		Maraviroc		0.94	0.17
		Fibrinogen		0.47	0.19
		Cocktail		2.28	0.45
	3i	Cocktail	Intact region	5.50	0.46
			Grafts	5.03	0.60
	4c	Wild type		0.083	0.009
		2 dps		0.135	0.024
		14 dps		0.161	0.030
		44 dps		0.163	0.025
	4d	Wild type		0.083	0.037
		2 dps		0.178	0.101
		14 dps		0.206	0.024
		44 dps		0.177	0.006
	4e	Wild type		0.060	0.032
		2 dps		0.203	0.045
		14 dps		0.147	0.023
		44 dps		0.153	0.035
		Wild type		0.117	0.042

Fig 4	4f	2 dps		0.173	0.040
		14 dps		0.180	0.054
		44 dps		0.234	0.022
	4i	Control		0.164	0.035
		Maraviroc		0.145	0.027
		Fibrinogen		0.172	0.034
		Cocktail		0.065	0.007
	4j	Control		0.177	0.026
		Maraviroc		0.163	0.056
		Fibrinogen		0.188	0.013
		Cocktail		0.064	0.010
	4k	Control		0.192	0.005
		Maraviroc		0.210	0.054
		Fibrinogen		0.179	0.042
		Cocktail		0.086	0.030
	4l	Control		0.541	0.072
		Maraviroc		0.458	0.049
		Fibrinogen		0.419	0.078
		Cocktail		0.334	0.050
Fig 5	5d	NPCs		4.23	1.12
		D7 neurons		1.81	0.68
		D60 neurons		0.47	0.21
	5k	vehicle + PBS		9.48	2.41
		vehicle + CCL3/4/5		28.76	3.71
		CCR5-RNAi + CCL3/4/5		9.84	1.84
		Maraviroc + CCL3/4/5		20.78	1.36
S fig 1	1a	Cocktail gel	pre	0.00	0.01
			0 day	0.32	0.09
			1 day	0.43	0.15
			3 day	0.64	0.15
			7 day	1.08	0.29
		Free maraviroc	pre	0.00	0.01
			0 day	5.01	0.11
			1 day	5.00	0.15
			3 day	4.94	0.07
			7 day	4.94	0.10
	1f	Brn2		26.25	1.43
		Ctip2		72.93	3.53
		Foxp2		51.81	6.97
S fig 2	2c	Control		0.00	0.00
		Maraviroc		0.00	0.00
		Fibrinogen		1.37	0.34
		Cocktail		12.30	3.18
S fig 3	3c	Ki67+		0.42	0.24
		Ki67-		99.58	
	3e	SOX9-		8.79	2.38
		SOX9+		91.21	
		Wild type	-14 dpt	389.14	18.196549
			pre transplantation	391.83	15.854618
			14 dpt	421.45	11.48217
			30 dpt	402.38	14.245728
		Sham	-14 dpt	413.95	13.52341
			pre transplantation	146.82	8.539021
			14 dpt	153.29	6.601554
			30 dpt	227.05	7.812416
		Control	-14 dpt	386.18	21.18456
			pre transplantation	124.95	9.154322
			14 dpt	163.41	7.547151

S fig 5	5a		30 dpt	272.65	14.054927
		Maraviroc	-14 dpt	369.67	17.24587
			pre transplantation	130.14	26.813544
			14 dpt	148.36	25.185436
			30 dpt	240.76	21.784138
		Fibrinogen	-14 dpt	400.54	24.583857
			pre transplantation	187.36	18.807264
			14 dpt	165.82	19.33546
			30 dpt	227.18	14.324168
		Cocktail	-14 dpt	372.91	25.64178
			pre transplantation	142.56	14.927521
			14 dpt	195.92	20.275216
			30 dpt	326.08	23.84214
	5b	Wild type	-14 dpt	4.92	2.6874201
			pre transplantation	6.74	1.963955
			14 dpt	8.11	2.4537121
			30 dpt	5.15	1.386493
		Sham	-14 dpt	6.52	3.42188
			pre transplantation	8.37	1.44574
			14 dpt	5.64	1.942385
			30 dpt	7.39	1.67484
		Control	-14 dpt	4.57	1.948335
			pre transplantation	26.16	9.154322
			14 dpt	20.54	6.64216
			30 dpt	15.86	3.21684
		Maraviroc	-14 dpt	6.94	1.41121
			pre transplantation	25.78	11.6524
			14 dpt	18.36	6.1548
			30 dpt	17.28	8.0495
		Fibrinogen	-14 dpt	5.91	3.18624
			pre transplantation	23.48	5.128301
			14 dpt	16.52	3.01512
			30 dpt	14.74	2.63319
		Cocktail	-14 dpt	7.66	1.42154
			pre transplantation	28.14	5.6218431
			14 dpt	15.92	2.60564
			30 dpt	9.58	3.403886
S fig 6	6b	Low CCL3/4/5		10.967735	4.349627
		High CCL3		41.737413	6.888689
		High CCL4		33.671553	8.556837
		High CCL5		32.429183	3.184713
		High CCL3/4/5		35.566413	13.41539
S fig 7	7b	vehicle + PBS		9.8572683	2.189782
		vehicle + CCL3/4/5		31.39621	5.197274
		CCR5-RNAi + CCL3/4/5		10.501137	1.563993
		Maraviroc + CCL3/4/5		20.777413	1.356111
